# Prevalence and Clinical Characteristics of Mycobacterial Diseases in the Barletta-Andria-Trani Province, Italy (2005–2013)

**DOI:** 10.1155/2016/9362708

**Published:** 2016-01-17

**Authors:** Gaetano Brindicci, Carmen Rita Santoro, Giovanna Trillo, Anna Volpe, Daniela Loconsole, Laura Monno, Tommaso Fontana

**Affiliations:** ^1^Operative Unit of Infectious Diseases, Vittorio Emanuele II Hospital, 76011 Bisceglie, Italy; ^2^Clinic of Infectious Diseases, Hospital-University Polyclinic, University of Bari, 70124 Bari, Italy; ^3^Department of Biomedical Science and Human Oncology, Hospital-University Polyclinic, University of Bari, 70124 Bari, Italy

## Abstract

Tuberculosis remains one of the major worldwide problems regarding public health. This study evaluates the burden of this disease in the BAT Province of the Apulia region (Italy); 12,295 patients were studied, including 310 immigrants. Tubercular disease and mycobacteriosis were found in 129 patients. The number of new TB cases/year ranged from three in 2005 to 12 in 2009. TB was more frequently localized in the lung (70.5%). 14.4% of cases were institutionalized patients for severe neurological and/or psychiatric disease. The database evidenced certain aspects of our study population: the large number of TB patients institutionalized between natives, but no larger presence of TB among HIV-positive patients in immigrants compared to Italians. Our findings should help to redefine the alarm regarding the spread of an epidemical form of TB but also to present certain criticisms regarding patient management (especially immigrants) regarding costs, hospitalization, and difficulty of reinstating the patient in the community. Further our data underscore the importance of prevalence of TB in bedridden, institutionalized patients.

## 1. Introduction

In spite of the fact that it is a preventable and curable disease, TB represents one of the major global health problems [1]; in fact in the 2013* Global Tuberculosis Report*, more than 8.5 million cases were identified, 1,450 million of which were fatal, thus indicating that TB is the most important infectious disease and cause of death after HIV [2]. In certain countries of Southern Africa (South African Republic, Lesotho, and Swaziland), the incidence of TB is greater than 1000 cases/100,000 inhabitants. In 2006, the World Health Organization (WHO) initiated the “*Stop TB Strategy*” with the objective of reducing, before 2015, both the prevalence and 50% mortality rate reported in the 1990 data and the global incidence of active TB, aiming to reach a goal of 1 case/100,000 million inhabitants by 2050 [2–5].

In Italy, TB is a relatively rare pathology and the 1998 incidence has been constantly less than 10 cases/100,000 inhabitants (considered by WHO as a low prevalence), which actually has even been reduced to less than 5 cases/100,000 [1]. However, beginning in the middle 1980s, an increase in TB has been reported, as in all the Western countries, due to the increase in risk categories (immunodepressed subjects, alcoholics, indigents, institutionalized patients, or those subjected to chemotherapy) and an increase of resistant strains to pharmacological therapies [6, 7]. In particular, the number of TB cases among immigrants has currently reached that of native subjects, especially in large cities [1].

Although most of the migrant population in Italy is concentrated in the north (about 60%), the increase of foreigners during 2011 was greater in the islands (Sardinia +13.7%) and Southern Italy (Puglia +13.5%) [8] than in the previous year. The most numerous foreigners are from Eastern Europe (Albania, Bulgaria, Ukraine, and Poland) and the Indian subcontinent (India, Bangladesh, and Pakistan), countries in which the epidemiological situation is complicated by the diffusion of mycobacteria resistant to at least two first-line antitubercular drugs (multidrug-resistant-TB, MDR-TB) or injected second-line drugs (extensively drug-resistant-TB, XDR-TB) [2].

In this study, we have attempted to quantify the presence of TB in the Italian BAT Province (Barletta-Andria-Trani) which has a population of about 400,000 inhabitants with a medium yearly increase of 0.1–0.3%. In recent years, a gradual yearly increase of 0.9% to 2.1% [9] in migrants from 2005 to 2010, respectively, has been observed; in fact, the number of non-Italian residents has more than doubled in 5 years (from about 3,600 to 8,440) compared to a steady decrease in the Italian population [10]. On Dec. 31, 2013, a total of 8,700 immigrants with regular permit to stay were present in the BAT territory (50% males) [9], equal to 2.1% of the entire population. Moreover, the economy of this province is based on agriculture, and it is reasonable to imagine the same number of migrants who were without regular documents (500 according to reliable estimates) was working in the fields.

The BAT Province includes 10 towns, three hospitals, and one Department of Infectious Disease in the Bisceglie Hospital. It is important to emphasize that Bisceglie is also the seat of a large private hospital (House of Divine Providence) with about 1,500 beds subdivided into an Alzheimer Unit, Rehabilitation Unit (Neurological and Cardiorespiratory), residence for cancer patients, and Psychiatric Clinic (in addition to Clinics for Acute, Neurological, Cardiac and Lung Patients). This Orthophrenic Institute (ex. Psychiatric Hospital of Bisceglie) which in the past provided 2,000 beds for institutionalized patients (from all of Italy) with cognitive-motorial problems either congenital or arising in childhood now includes 700 beds.

Infectious disease clinics have also become important observers of most TB cases notified for a series of reasons (possibility of isolation, association with HIV and immunosuppression). The Clinic of Infectious Diseases of the Bisceglie Hospital is one of the largest units providing the largest number of beds throughout Italy.

## 2. Materials and Methods

Our study includes all hospitalized patients at the Unit of Infectious Diseases of the Bisceglie Hospital between Jan. 1, 2005, and Dec. 31, 2013, subjected to a retrospective analysis. The database includes demographic (age, sex, residence, and nationality) and clinical (diagnosis at admission and dismissal, length of hospitalization, and mode of dismissal) data in addition to fiscal code, temporary presence code, or nonregistered European [11] code. Regarding dismissal diagnosis, the database also provides the principal and secondary diagnoses (up to five) reported in the hospital dismissal form (SDO).

Italy legally requires all citizens to be inscribed in the National Health System or to be classified as a* foreigner temporarily present* or* nonregistered European*, thus guaranteeing health assistance also for foreigners without a regular residence permit. This is a constitutional privilege and the legislative degree number 286 of July 25, 1998, regards regulations for foreigners [11].

Selection of cases was based on the presence of a TB diagnosis (and/or mycobacteriosis) according to the “International Classification of Diseases-IX-Clinical Modification” (ICD-IX-CM) [12]. In the case of mycobacteriosis disease, the code defined the site of disease (between 0010 and 0018 for TB and 031.0 and 031.9 for nontubercular mycobacteriosis, NTM).

### 2.1. Statistical Analysis

Data were processed using the software package STATA 11.0. *χ* ^2^-test was used to compare proportions. For small samples, Fisher's exact test was used. *t*-test was used to compare means. If a *p* value was below 0.05, the difference between proportions was considered to be statistically significant.

## 3. Results

A total of 12,295 patients (including 310 foreigners) were hospitalized at the Infectious Diseases Unit of the Bisceglie Hospital from 2005 to 2013 (70% males) with a median age of 48,3 years and mainly residing in the BAT (68.9%) and Bari (28.9%) areas. Over this nine-year period, the number of patients hospitalized was fairly constant (1,350 yearly, 1,444 in 2007 to 1,308 in 2011). However, the number of migrants almost tripled (20 in 2007 and 59 in 2012). The length of hospital stay ranged from 0 to 214 days with a median of 29 days.

The nosological code referring to TB and mycobacteriosis was found 159 times, that is, 124 times as the principle diagnosis and 16, 8, 7, 4, and 0 times for the five fields of secondary diagnosis in the dismissal form (SDO). TB was diagnosed in 149 patients (93.7%) and the remaining 10 cases were NTM. In thirty cases the same patients were admitted several times in our unit and, therefore, these cases were not considered.

Of the 129 TB cases, 85 were Italians and 44 were immigrants. The median age of TB patients was 48.3 years (range 16–88) and 70% were males. The average age of immigrant TB patients was 36 years (standard deviation ±12.9, range 18–82) and was significantly reduced (*p* < 0.0001) compared to that of the TB Italian patients (51 years, SD ±19.01). The 66% of immigrant patients with TB were males (29/44). The Italian TB patients residing in the BAT Province were 65/85 (76%), in particular Bisceglie had 33, Barletta 11, Andria 10, Trani 8, Canosa 2, and Trinitapoli 1, while those living in the other provinces included Bari (13 patients, 15.2%), Foggia (4 patients, 4.7%), and Lecce, Brindisi, and Rome (1 patient each).

**Figure 1:**
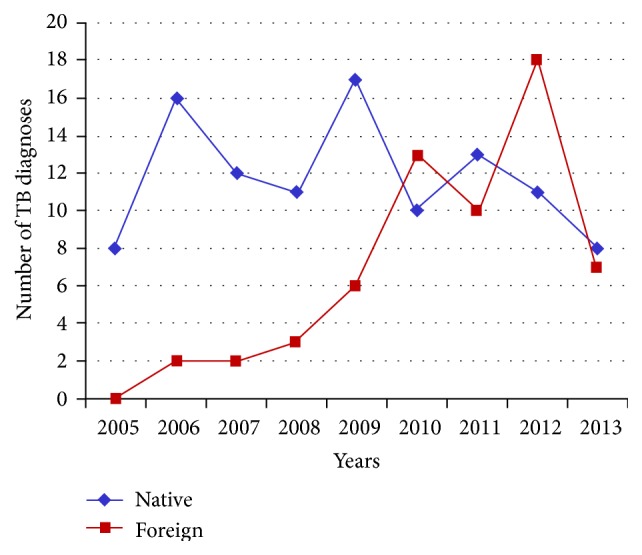
Patients admitted with tuberculosis in the Infectious Disease Unit of Bisceglie (2005–2013).

**Figure 2:**
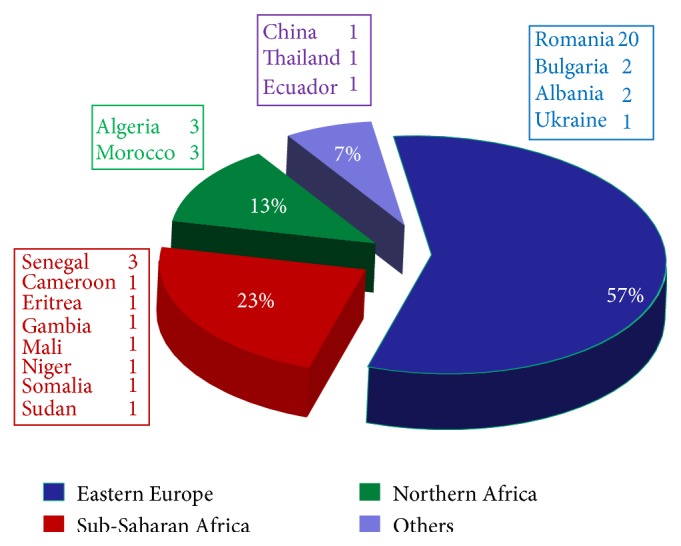
Countries of origin of the 44 immigrants with TBC.

The migrants with TB living in the BAT Province were 25/44 (56%), in particular Andria had 10, Bisceglie, Barletta, and Trani 4 each, Canosa 2, and San Ferdinando di Puglia 1, followed by 1 each for the Province of Foggia (9/44), Bari (8/44), Brindisi, and Lecce.  Table 1 shows the comparison of Italian and immigrant patients regarding epidemiological and clinical characteristics. For the major part, the migrants originated from Eastern Europe (25/44, 22.7%, 80% Romanian nationality), Sub-Saharan Africa (10/44, 22.7%), and Northern Africa (6/44, 13.6%) (Figure 2). Of the 44 immigrant patients, 15 (34%) were illegally present in this area and possessed or furnished when admitted a STP code (temporary residence form) or an ENI (nonresident European) code. A total of 8/15 immigrants were Romanian and the other seven were from Northern Africa (2 Algerians, 2 Moroccans) or from Eastern Africa (Eritrea, Somalia, and Sudan). The number of new TB cases in immigrants varied from 2 (in 2006) to 18 (in 2012) and the median was 4.8 cases/year, increasing constantly and exceeding 50% of total cases in 2012 (Figure 1). The prevalence of disease in the entire BAT Province was of a median of 1.8/100,000 inhabitants/year (range 0.75 to 3/100,000 inhabitants/year).

**Figure 3:**
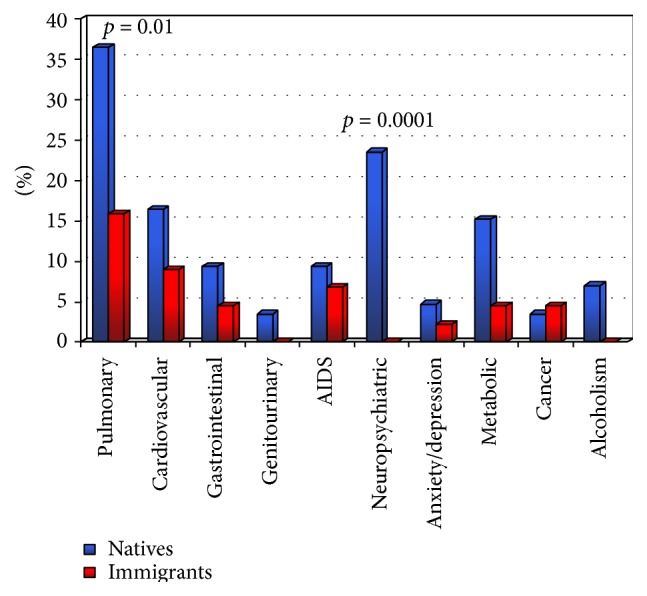
Comparison of comorbidities (%) between natives and immigrants.

Regarding TB clinical characteristics, the most common site was the lung (91/129, 70.5% of cases) followed by lymphoglandular (8 cases), disseminated (8), and bone (4) sites. Also in immigrants, the most frequent site was the lungs (38/44, 86%), followed by the disseminated, bone (2), and lymphoglandular (1) forms; lastly, one case of pulmonary mycobacteriosis was found in a Chinese patient. Among the TB patients, 20% presented a comorbidity involving the respiratory system (chronic bronchial obstruction, pulmonary fibrosis, and pulmonary hypertension), 13.8% regarded the cardiovascular system, and 7.5% regarded the gastrointestinal apparatus. A total of 8.5% of patients were HIV positive (11 patients, only 3 immigrants) while 14.4% (22 Italian patients) were hospitalized due to serious neurological and/or psychiatric problems (mental retardation, schizophrenia, results of cerebral ictus).  Figure 3 presents the comorbidities of the Italian and immigrant TB patients in a more detailed manner.

The Italian TB patients were hospitalized for a median of 29 days (range 1–213); those dismissed in a suitably improved condition were 105/129 (61.4%) while 3.1% were transferred to a ward for acute patients and 9.3% (12/129) voluntarily left the hospital against the advice of the physician, 3/12 of them within 14 days after recovery; 6% of cases died, equal to 0.22/100.000 inhabitants/year. The length of recovery among immigrants ranged from 4 to 120 days with a median of 29 for legal and 31 days for illegally present immigrants. Those dismissed in a better state of health were 79.5% (35/44) while 4.5% were transferred to other wards and 13.6% of patients (6/44) left the hospital against the advice of the physician.

## 4. Discussion

Our data confirm certain characteristics previously reported in the literature (prevalence of TB localized in the lung in males and in young immigrants) [2, 13, 14] which will not be repeated here. The total number of cases demonstrates that, even today, tuberculosis is a disease present in Italy, even if not frequently. If we consider that the prevalence curve of this disease began to increase in Western countries over a period of hundred years, it then seemed to return to zero. However, new cases of TB then reappeared again often due to an incorrect management of health services (e.g., in the 1980s it was not only AIDS to determine this increase in TB, but also the lack of prevention campaigns and elimination of public health structures [15]).

In the BAT Province, 1.2% of all recoveries in the Infectious Disease Unit regard mycobacteria. This is apparently a small number with a tendency, however, to increase due to both the migratory phenomenon (as in all provinces with a large presence of immigrants) and the increase of pharmacologically induced immunodeficiencies. Considering that the population of our province reaches 400,000, our data indicates a TB prevalence of 1.8 cases/100,000 inhabitants, in accordance with data previously reported for the entire country. At present, in Italy, the cases of TB among immigrants have continually increased; in 1995, 525 cases were noted (10% of total amount) which expanded to 2,026 cases in 2008 (46% of total) [9, 10]. This variation appears to be due to the numerical increase of the foreign population (from 700,000 in 2002 to more than 5 million in 2013) [4]. Therefore, the TB epidemic appears to be changing even in our relatively small province and is beginning to assume the characteristics described on the national level [11].

The inversion evidenced in 2012 regarding the increase in number of TB diagnoses in immigrants compared to that found for Italians can be explained by the fact that from the year of establishing this province (2005) no increase in Italian residents has been noted while there was a threefold increase in the migrant population; in fact, Puglia has become the region with the highest increase (15%) between 2012 and 2013.

When considering comorbidity, certain interesting characteristics also appeared. The 8.8% prevalence of AIDS found in our cases was comparable to that of European [16] and Italian [17] data even if, surprisingly, it was found to be less among immigrants who also demonstrated a lesser dependence on alcohol and presence of neuropsychiatric problems. It must be remembered that psychic disturbances (also posttraumatic problems related to stress) are well known events which are quite important according to the ISTAT annual reports (10% of foreigners) [9]. Only disturbances of respiratory (65%), gastrointestinal (20%), osteomuscular (17%), and nervous (20%) systems present greater percentages, while only 2.6% of migrants were also affected by infectious diseases. These results can be partially explained both by the fact that migration to the BAT Province is a relatively recent phenomenon and by the fact that diagnosis of HIV/AIDS in migrants (as in the rest of the country) is generally delayed, resulting in higher frequency of late presenters in this group [18, 19].

The greater prevalence of patients with serious disabilities and those who were hospitalized merits a closer analysis. Our study included 22 hospitalized patients (all Italian), eight of them with serious mental retardation, five with epilepsy and five with Down syndrome, three with schizophrenia or serious psychoses, two with damage from cerebral ictus, and one with congenital tetraplegia.

It is well known that hospitalization presents a risk factor for TB and the Ministry Guidelines indicate that all elderly subjects living in rest homes or submitted to lengthy recoveries are classified as risk categories. However, our study emphasizes that the young age of our patients recovered at Bisceglie Hospital was most likely due to the fact that these patients had been hospitalized, even at a young age, in the Divine Providence hospital (median age 62,5 years; 12/22 were younger than 65), and the prolonged presence in this hospital was the risk factor for them.

The length of hospitalization (a median of 29 days for TB) was much longer compared to the median of other hospital recoveries in the same department (9 days) [20]. However, due to the number of voluntary dismissals, especially among immigrants, the length of time was probably less. In fact, the cases of patient dismissal within the first 15 days against the doctor's opinion were 5.6%, a high percentage when considering that these are all contagious patients. Moreover, the length of hospitalization also has repercussions on the cost as TB patients require up to three times that of most patients (up to a maximum of 20–25 times) and the length of isolation must also be taken into consideration. The medium length of recovery, which may extend up to a maximum of 213 days, not only reflects the natural history of the disease, but also is due to the caution of the physician when the patient lacks familial and social assistance during the induction and maintenance phase of therapy at home. This data is important for indigents, homeless immigrants, and institutionalized patients (for whom the importance of directly observed therapy and entrance in a secure residence is easily understood); these data determine a* boomerang effect* (for first-aid emergency workers and those in other hospital wards) not only regarding health costs but also due to the difficulty to find beds “in isolation” when a suspected case is discovered.

The Guidelines proposed by the Ministry of Health state that “the TB patient is often hospitalized without motivation and for longer periods of time than absolutely necessary…” and that “… hospitalization is indicated in case of: extensive disease or tubercular meningitis, compromised clinical conditions, immune compromised patients, positive bacteriological expectorants when a safe isolated home is not available, and presence or suspected polyresistence [*sic*] …” [21]. However, often these indications clash with daily life and it is impossible for the physician to be sure about proper isolation from those with whom they mix (the homeless, indigents, and alcoholics).

## 5. Conclusions

1. Our data should help to place the alarm regarding the spread of TB in the mass media into its proper prospective indicating several crucial aspects concerning the access and use of health services by legal and illegal immigrants and how these factors determine correct patient management.
2. The promotion of research programs would be extremely useful to identify symptomatic TB cases, not only in countries with a high incidence of disease as previously stated [22] and indicated by WHO. Further research is needed to also identify those categories of patients at risk and rarely encountered, thus reaching an early diagnosis in a higher percentage of patients and providing an improvement in health for the general population and a reduction of costs for the national health system.

## Figures and Tables

**Table 1:** Epidemiological and clinical differences between native and immigrants patients with TBC.

	Natives (85 pts.)	Immigrants (44 pts.)	*p* value
Average age at admission SD	51.6 ±19.01	35.7 ±12.9	
With regular permit to stay		33	*p* < 0.0001
Without regular permit to stay		37	
% of male sex	70	66	
% of BAT residents	72.9	56.8	
% of pulmonary localization	63	86	*p* = 0.04
% of AIDS	9.4	6.8	NS
% of pulmonary comorbidity	36.5	15.9	*p* = 0.01
% of institutionalized patients	23.5	0	*p* < 0.0001
Average length of stay	30	29	
With regular permit to stay		29	
Without regular permit to stay		31	
% of voluntary dismissals	7.4	13.6	
